# Supplementary material for: Dynamics of soliton self-injection locking in optical microresonators
Source: Nat Commun. 2021 Jan 11;12:235. doi: 10.1038/s41467-020-20196-y (PMC7801488; doi:10.1038/s41467-020-20196-y)
Supplement: Supplementary file 1 — Supplementary Information [file 41467_2020_20196_MOESM1_ESM.pdf]

Supplementary Information – Dynamics of soliton self-injection locking in optical microresonators

Andrey S. Voloshin, Nikita M. Kondratiev, Grigory V. Lihachev, Junqiu Liu, Valery E. Lobanov, Nikita Yu. Dmitriev, Wenle Weng, Tobias J. Kippenberg, and Igor A. Bilenko

SUPPLEMENTARY NOTE 1: LINEWIDTH OF SIL DFB

The employed DFB laser diode has the wavelength of 1547 nm, the maximum optical output power of ~ 120 mW at the injection current of ~ 472 mA and the temperature of 20 C°. Such high-power DFB laser diodes with similar characteristics have been reported previously [1–4], and are commercially available. The spacing between suppressed modes is 24.5 GHz, defined by the cavity length of the laser diode. Heterodyne measurements determine the instantaneous linewidth in the free-running regime with a reference laser (TOptica CTL), and is estimated as 119 kHz using a Voigt profile. The laser diode is installed in a custom-made mount, providing good heat sinking, but it may also be placed on a submount in a conventional butterfly package.

Compared with the 119 kHz Lorentzian linewidth of the free-running DFB diode, the fitted Lorentzian linewidth (using a Voigt profile) of the SIL DFB is reduced down to 1.1 kHz, as shown in Figure 3(c) in the main text. Using the parameters such as the linewidth of the free-running DFB laser diode $\delta\omega_{\mathrm{free}}/2\pi = 119$ kHz, the quality factor of the DFB laser cavity $Q_{\mathrm{LC}} = 10^4$, and the linewidth enhancement Henry factor $\alpha_g = 2.5$ [1, 8], the theoretical linewidth of the SIL DFB is calculated as $\delta\omega_{\mathrm{locked}}/2\pi = 0.23$ Hz. Note that this value $\delta\omega_{\mathrm{locked}}/2\pi$ does not take into account other noises, and is thus much smaller than the measured value of 1.1 kHz. We attribute the linewidth broadening to the resonance frequency fluctuations, estimated to be in the Hz-level [9, 10], and mechanical instability of our setup, estimated to be in the kHz-level[11].

The self-injection locking range is defined as the difference between the maximum and minimum laser frequency when laser SIL is maintained. It can be estimated for the linear regime [11] as $\Delta\omega_{\mathrm{lock}}/2\pi \approx r\sqrt{1+\alpha_g^2}\omega_0/Q_{\mathrm{LC}}/2\pi = 12.4$ GHz, and the experimentally measured value is more than 7 GHz. The discrepancy is obviously the result of high power as according to the nonlinear theory the locking range tends to reduce with input power (see Supplementary Figure 2(c)). Note that increasing the locking range $\Delta\omega_{\mathrm{lock}}/2\pi$, e.g. by increasing the microresonator Q, may lead to the overlap of the locking ranges of two neighboring resonances. The locking range overlap can lead to mode competition and an unstable SIL regime.

Also a special multi-frequency regime of lasing [5] is studied and mutual coherence of different laser lines is measured. When the backscattered laser radiation from the microresonator is strong enough, it may lead to a spatial hole burning (SHB) effect. The spatial structure created in the active media of the semiconductor diode may play a role of spectral selective element, so-called, self-induced grating. Therefore, the spectral selectivity of the DFB laser diode is determined not only by the DFB structure but also by the SHB structure. The influence of SHB may be stronger than the DFB structure, and the latter ceases to be a major spectral selectivity element. In this case, the DFB laser diode may operate in multi-frequency regime (Supplementary Figure 1)). In other words, the side-mode suppression ratio of the DFB laser diode structure is significantly decreased. Such a multi-frequency regime was observed in our experiment. It should be noted that the spectral profile strongly depends on the optical feedback level. Another explanation may be given by period-one (P1) oscillations in semiconductor lasers [6, 7]. Multi-frequency regime of operation generates a powerful RF beatnote signal of 32 different spectral optical components. We measure SIL DFB optical line's mutual stability in such a multi-frequency regime as 3.8 kHz.

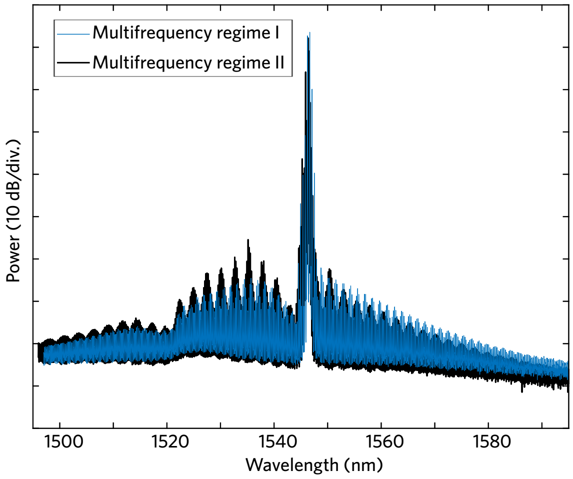

Supplementary Figure 1: Multi-frequency regime of DFB diode operation with different feedback levels (regime I and regime II).

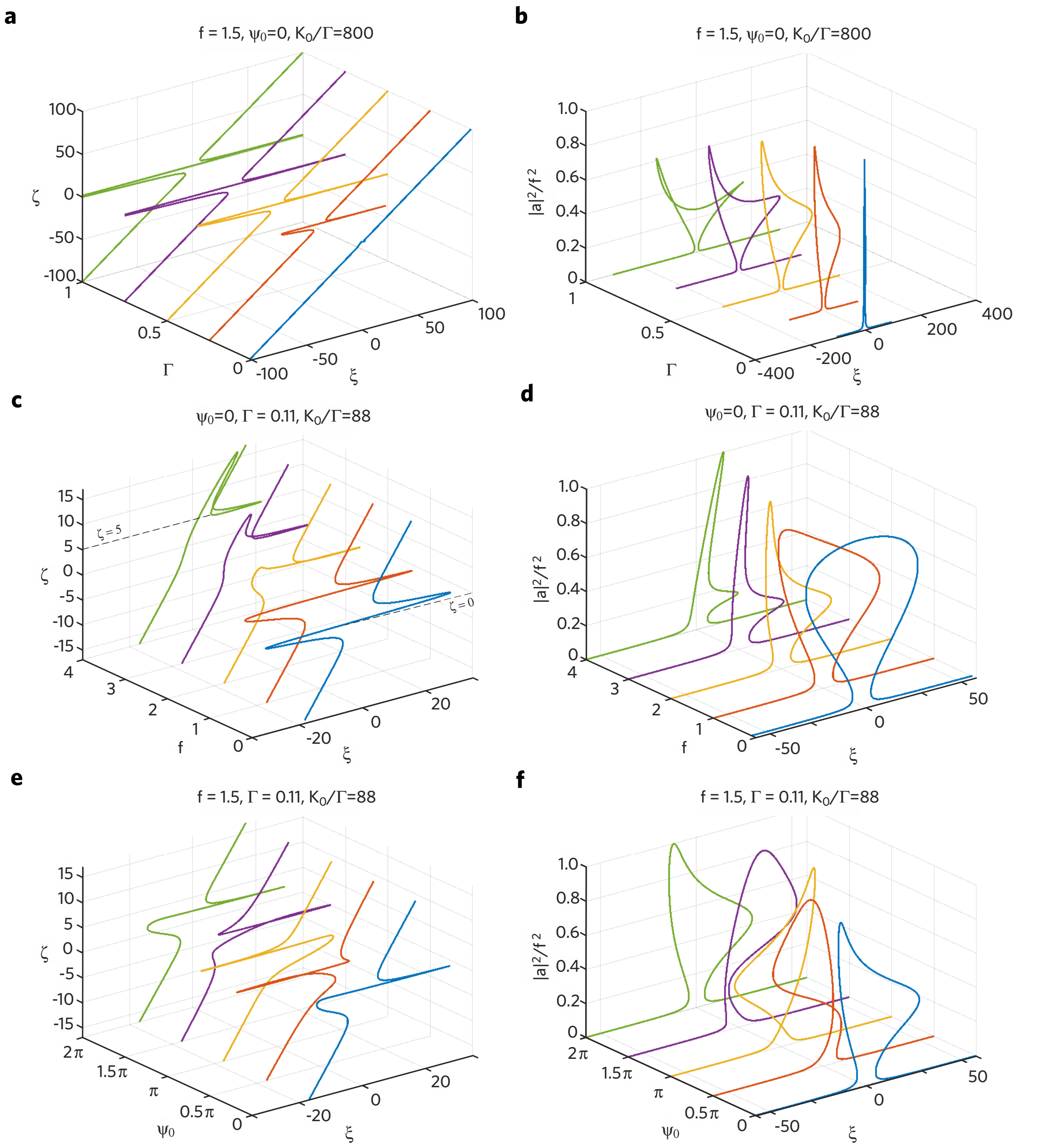

Supplementary Figure 2: Different regimes of laser self-injection locking to nonlinear cavity. (**a**)-(**b**): Dependence on backscattering Γ. Higher mode-coupling parameter Γ provides wider locking range and twists the resonance curve. (**c**)-(**d**): Increasing of the pump power f leads to the shifting of the tuning curve to the red-detuned region, as necessary for the soliton formation, but reduces the locking range. (**e**)-(**f**): Influence of the optical locking phase ψ_0 on the tuning curve. Changing the phase by π effectively mirrors the curve over $\xi = \zeta$ in ξ coordinate.

SUPPLEMENTARY NOTE 2: DIFFERENT REGIMES OF LASER SELF-INJECTION LOCKING TO NONLINEAR CAVITY

We study how the nonlinear self-injection locking depends on significant parameters: locking phase (phase delay between the laser cavity output and the optical feedback), laser diode power, and the normalized mode-coupling parameter Γ. The nonlinear tuning curve $\zeta(\xi)$ and the intracavity power $|a(\xi)|^2$ are presented in Supplementary Figure 2 left and right columns correspondingly. The first row in Supplementary Figure 2 (panels a and b) shows how the tuning curve changes with increasing of the normalized mode-coupling parameter Γ from 0 to 1. First, $\Gamma \approx 0$, and there is no optical feedback and no self-injection locking. The tuning curve is linear ($\zeta = \xi$), and the resonance curve is Lorentzian. Then while Γ increases, the locking range increases too. Laser tuning curves are asymmetrical, because the pump power $f = 1.5$ is high enough for the nonlinearity manifestation.

The second row (panels c and d) shows the dependence on the normalized pump f. First, f is small, and we observe linear self-injection locking [11]. The tuning curve becomes asymmetric with higher f and the locked state (the nearly horizontal part of the tuning curve) shifts to higher detunings ζ, making soliton generation possible. Note that the locking range decreases with the pump power. The intracavity power $|a(\xi)|^2/f^2$ looks very different from the conventional nonlinear resonance in the presence of the optical feedback and both curves become asymmetric already at $f = 1$. Note that for high input power, the intracavity power is similar to the nonlinear resonance with the backward wave [12].

The third row (panels e and f) shows the dependence on the locking phase ψ_0. The tuning curve drastically changes with this parameter, the most sensitive in our experimental setup. It depends on the distance between the laser cavity and the microresonator, its change for 1.5 μm corresponds to the change of the phase by 2π.

SUPPLEMENTARY NOTE 3: OPTIMAL LOCKING PHASE AND ITS CONTROL

We observe the dependence of the microresonator transmission on the optical alignment (the distance between the laser facet and the chip with microresonator) experimentally and thus the locking phase ψ_0 (see Supplementary Figure 2(e,f)). The transmission trace changes drastically with different locking phases and is very different in forward and backward scans.

Note that the trace "Optical phase V" in Supplementary Figure 3 does not contain the high-Q resonance. There are some resonances with low Q but with strong back-reflection. Such resonances appear due to a Fabry-Perot cavity formed by Si_3N_4 photonic chip edges. Such edges are comfortable for butt-coupling, but they cause a strong influence on the laser diode. In some cases, the high-Q resonances are hindered because the laser diode is locked to the Fabry-Perot resonances. This undesired back-reflection may be suppressed by introducing angled waveguide tapers.

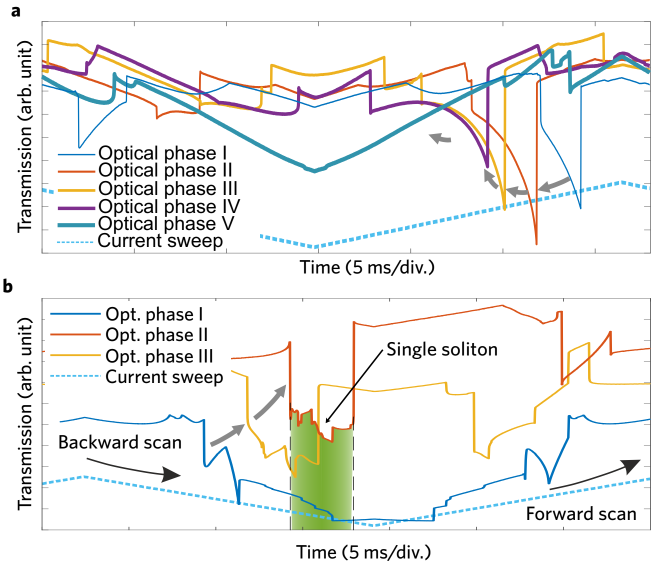

Supplementary Figure 3: Experimental dependence of the transmission power on the optical locking phase ψ_0.

SUPPLEMENTARY NOTE 4: DIFFERENT MICROCOMB STATES IN LASER SELF-INJECTION LOCKED REGIME.

In our experiments, a variety of optical spectra of microcombs generated in the Si_3N_4 microresonator is observed in forward and backward scans. We scan the laser diode frequency with injection current over the nonlinear resonance and observe the formation, first, of a modulation instability (characteristic primary combs in Supplementary Figure 4(a)), then a chaotic comb (Supplementary Figure 4(b)) with a flat-top symmetric envelope [13]. Tuning further we observe breather solitons, soliton crystals (Supplementary Figure 4(c)), single and multi-soliton states (Supplementary Figure 4(d)). Other observed comb states are shown in Supplementary Figure 4(e)-(h).

SUPPLEMENTARY NOTE 5: PHASE NOISE CHARACTERIZATION

Previously, the phase noise of soliton repetition rates has been characterized only in bulk crystalline microresonators [14, 15] and silica microdisks [16, 17], because the repetition rates are electronically detectable. This has not been possible previously in any integrated platform, including Si_3N_4, as integrated solitons of microwave repetition rates have not been demonstrated [18–20] due to

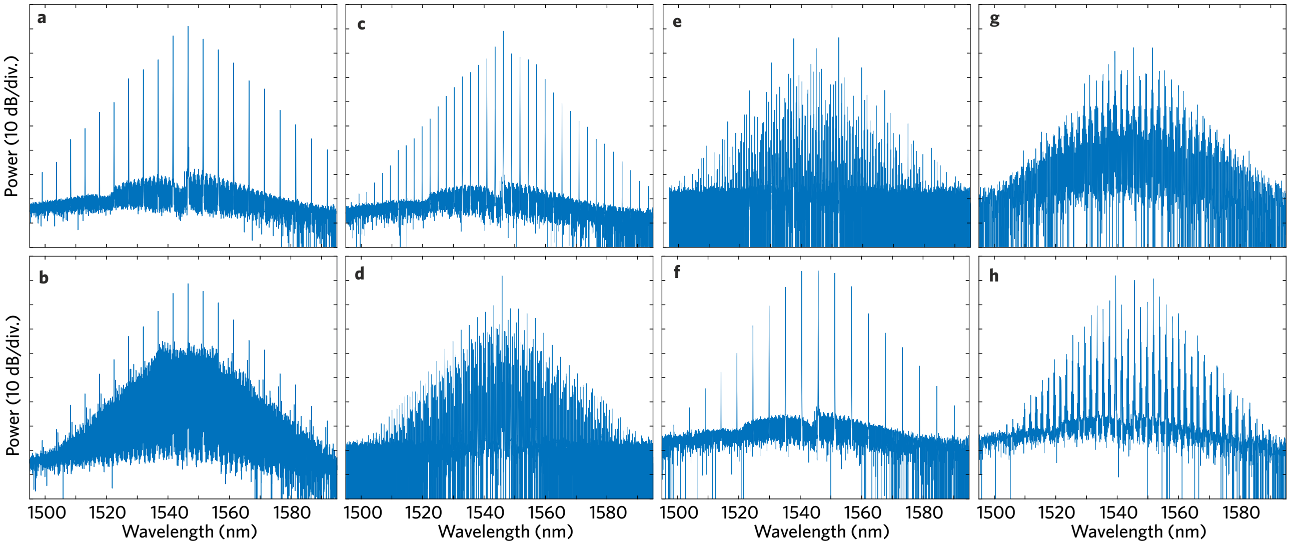

Supplementary Figure 4: Different microcomb states in laser self-injection locked regime.

the limited microresonator Q-factors. Recent advancement in the fabrication of ultralow-loss Si_3N_4 waveguides [21] has allowed the generation of single solitons with repetition rates in the widely employed microwave K- and X-band [22]. Equally important, the phase noise of the integrated soliton microcombs has been characterized and compared to the fundamental limit imposed by Si_3N_4 thermo-refractive noise [10].

First, we performed a measurement of the phase noise of laser diode in different regimes using heterodyne technique and IQ-data measurements. In RF measurements, heterodyne signal represented by a sinusoid with angle modulation can be decomposed into, or synthesized from, two amplitude-modulated sinusoids that are offset in phase by one-quarter cycle ($\pi/2$ radians): $A\cos(2\pi ft+\phi) = I\cos(2\pi ft) - Q\sin(2\pi ft)$. All three functions have the same center frequency. The amplitude modulated sinusoids are known as the in-phase (I) and quadrature (Q) components. This approach is very convenient for RF signal processing. Frequency and phase noises may be extracted from IQ-data as periodogram power spectral density or be found using Welch's overlapped segment averaging estimator. Due to the fact that the 30 GHz carrier frequency was out of the detection bandwidth of our ESA (26 GHz), the signal was down-converted with an RF mixer and a local oscillator (Keysight N5183B) $f_{LO} = 20$ GHz, in order to facilitate the detection. The 35 GHz soliton beatnote signal was detected directly.

We measured the phase fluctuations of 30 GHz and 35 GHz soliton repetition rates in the SIL regime. The measured phase noise of the 35 GHz single soliton repetition rate, shown as the purple curve in Figure 2(d) of the main text, has single-sideband phase noise of -70 dBc/Hz at 1 kHz Fourier offset frequency, -96 dBc/Hz at 10 kHz, and -113 dBc/Hz at 100 kHz. The detector shot noise for the studied signal is -144 dBc/Hz.

Different soliton states demonstrate different phase fluctuations, another 30 GHz soliton state (red curve) provides the phase noise -65 dBc/Hz at 1 kHz, but much higher phase fluctuations -88 dBc/Hz at 10 kHz and at higher offsets. The ability to switch between soliton states and different phase noise for different soliton states indicates that in our experiment the phase noise characteristic might be further improved.

Thus SIL microcomb provide competitive phase noise characteristics within 1 kHz - 1 MHz in comparison with breadboard implementation [22]. As a future investigation, we need to understand why self-injection locking does not suppress phase noise at low frequencies (below 1 kHz). Also, robust packaging of integrated microcomb sources may decrease the phase noise at these frequency offsets. Then we need to ensure that "quiet point" [16] is accessible in the self-injection locking regime.

SUPPLEMENTARY NOTE 6: TEMPORAL WAVEFORM OF SOLITON PULSE

Soliton microcombs are a train of ultra-short pulses, but direct observation of such temporal solitons is challenging. These pulses have widths of hundreds of femtosecond and repetition rates from GHz to THz. The problem is that optical spectrum measured by OSA is the Fourier transform of the auto-correlation function (ACF). But AFC may exhibit narrow spikes, that are easily confused with pulses, even in the case where no pulses are present in the cavity. It may happen if all phases between comb lines are constant in time but arbitrary. This results in a periodic, modulated time domain output from the microresonator but not the truly pulsed behavior (see Supplementary Materials of [23] for more

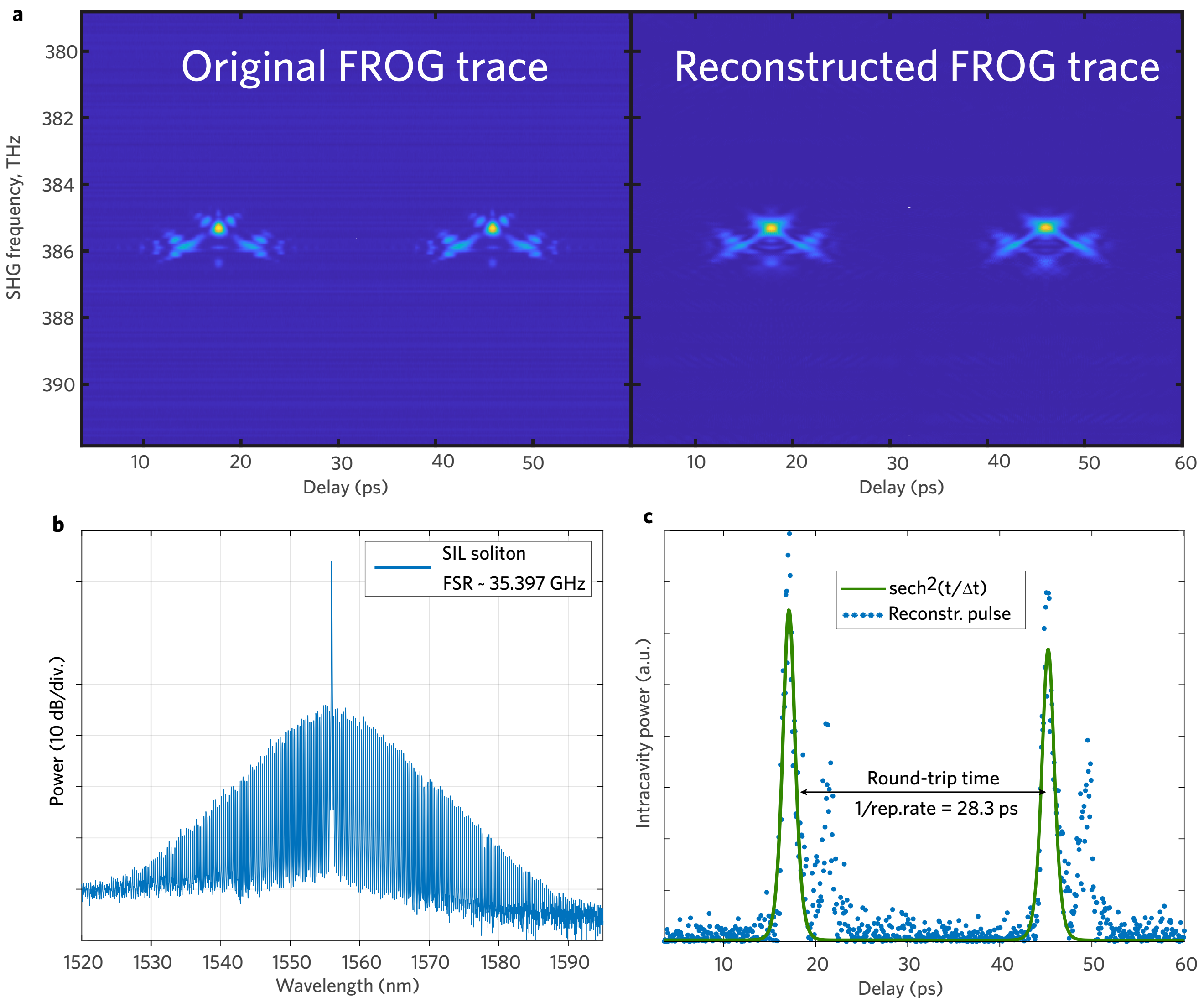

Supplementary Figure 5: **Frequency Resolved Optical Gating (FROG) of self-injection locked soliton.** **(a)** Original FROG trace measured for soliton microcomb with a repetition rate of 35.397 GHz is on the left panel. The microcomb was, first, filtered by using fiber Bragg grating to suppress the central line and amplified to ≈ 100 mW by using cascaded optical amplifiers (with non-flat gain). The fiber Bragg grating filtered not only the central comb line but also neighbor lines because its bandwidth is more than the comb lines spacing. This caused the changes in the optical spectrum but does not affect the phase relations between comb lines and pulsed waveform. That is why the FROG trace does not correspond to the pure single soliton temporal profile, but still exhibits the optical pulse with a width of less than 1 picosecond. The reconstructed FROG trace is on the right panel. **(b)** Optical spectrum of 35.397 GHz self-injection locked soliton. **(c)** The reconstructed intracavity power of the soliton pulse. Deviation and broadening from the expected soliton temporal profile are caused by the cascaded amplification by 2 non-flat gain optical amplifiers and filtering using a fiber Bragg grating.

details).

The first Frequency Resolved Optical Gating (FROG) measurement of soliton microcombs in microresonators was presented in [23] and demonstrated that soliton microcombs are optical pulses, separated by the cavity roundtrip time with constant pulse amplitudes, as expected for temporal dissipative solitons [23]. Since this research, it is widely accepted to demonstrate two measurements to prove existence of soliton pulses in microresonators (with anomalous dispersion): the sech^2 envelope of the optical spectrum and of the background noise (the RF noise from DC to 1-5 GHz). Narrow-band beatnote signal at the soliton repetition rate frequency is a good addition to the soliton state, but it impossible to measure

it for most photonic chip-based microresonators, because their FSR exceed 100 GHz. In our experiments, we observe sech^2 spectrum, zero background noise, and ultra-narrow beatnote signal. That allows us to conclude that we study temporal soliton.

To provide one more evidence of soliton origin we perform FROG measurement of integrated optical microcomb. We generate self-injection locked soliton in a microresonator with FSR of 35.397 GHz. This single soliton provides pure spectral characteristics and has a power of less than 1 mW. Before the FROG setup, the optical spectrum of self-injection locked soliton is filtered by a fiber Bragg grating (FBG) for pump suppression (−30 dB) and amplified to ≈ 100 mW by using cascaded optical amplifiers. This caused the changes in the optical spectrum but does not affect the pulse origin of the soliton. The intracavity field distribution reconstructed from the FROG trace proves that the studied spectrum corresponds to the ultra-short bright soliton (see Supplementary Figure 5).

SUPPLEMENTARY NOTE 7: OPTIMIZING COMB POWER

In soliton regime the pump-to-comb conversion efficiency is not very good. However it can be optimized to some extent. Using the formula for the comb envelope [23] we get the following:

$$P_\mu = \eta\kappa \frac{n_{\rm eff} n_{\rm eff_c} D_2 V_{\rm eff}}{4\omega c n_2} \mathrm{sech}^2\left(\frac{\pi}{2}\sqrt{\frac{D_2}{\kappa\zeta}}\mu\right), \qquad (1)$$

where $n_{\rm eff}$ and $n_{\rm eff_c}$ are effective refraction indices of the WGM and the coupler mode, n_2 is the nonlinear index of the microresonator, D_2 is the second order dispersion coefficient, η is the pump coupling coefficient, κ and ω are the loaded linewidth and eigenfrequency of the WGM and $V_{\rm eff}$ is its effective volume. The μ is the mode number, counting from the pumped mode and the formula is valid only for $\mu \neq 0$ (the zero mode should take the interference with the pump into account). The ζ is the effective detuning. The WGM cross section area is assumed the same as for the output beam.

This formula provides insight into both the comb width and comb tooth power. First, the power can be increased by increasing the effective detuning ζ, which also leads to the comb width increase. However, this parameter has finite borders (see Eq. 1 in the main text) in the unlocked state but the maximum can be increased with the input power. Furthermore, its change is difficult in the locked state as the effective detuning is fixed and exhibits a complicated dependence on the pump power, microresonator backscattering rate and quality factor. Second, the sideband power can be increased by means of the dispersion engineering. It is worth noting, that, the optimum over D_2 exists, so if the power is important, it should not be decreased abruptly. Last, the coupling increase also allows to increase the sideband power. However this can decrease the loaded quality factor, increasing the power threshold for the comb generation.

To maximize the power of the weakest comb lines, the group velocity dispersion (GVD) and the coupling rate of the microresonator to the bus waveguide may be varied within values that can be engineered by varying the Si3N4 waveguide dimensions as well as the resonator-to-waveguide distance in combination with specially designed coupling regions [24]. Another approach for power enhancement is the use of normal GVD regime, which supports formation of dark soliton pulses. These temporal localized structures provide much higher pump-to-comb conversion efficiency [25] up to 41% [26]. The proposed in the main text theoretical model of nonlinear self-injection locking is valid for this regime. Moreover, our recent numerical studies suggest that the SIL allows for solitonic pulse generation in normal GVD regime without any additional efforts [27].

Nevertheless, we demonstrated the self-injection locked soliton with power of 1.4 mW. The comb optical spectrum (Fig. 3b of the main text) consists of more than 30 lines with power of -20 dBm. The one of the record transmision data experiments, where transmission of a data stream of more than 50 terabits per second, utilizes the Kerr microcomb with 179 lines with power higher than -20 dBm [24].

SUPPLEMENTARY REFERENCES

[1] Piprek, J. & Bowers, J. E. *Analog modulation of semiconductor lasers*, 57–80 (Cambridge University Press, 2002). URL https://doi.org/10.1017/CBO9780511755729.004.

[2] Faugeron, M. *et al.* High-power, low RIN 1.55-μmdirectly modulated DFB lasers for analog signal transmission. *IEEE Photonics Technology Letters* **24**, 116–118 (2012).

[3] Zhao, Y.-G., Nikolov, A. & Dutt, R. 1550 nm DFB semiconductor lasers with high power and low noise. In Witzigmann, B., Henneberger, F., Arakawa, Y. & Freundlich, A. (eds.) *Physics and Simulation of Optoelectronic Devices XIX*, vol. 7933, 525 – 531. International Society for Optics and Photonics (SPIE, 2011). URL https://doi.org/10.1117/12.873973.

[4] Takaki, K. *et al.* High-power CW-DFB LDs for optical communications. *Furukawa Review* **23**, 1 – 5 (2003). URL https://www.furukawa.co.jp/review/fr023/fr23_01.pdf.

[5] Rabinovich, W. S. & Feldman, B. J. Spatial hole burning effects in distributed feedback lasers. *IEEE Journal of*

Quantum Electronics **25**, 20–30 (1989). URL https://doi.org/10.1109/3.16236.

[6] Simpson, T. B., Liu, J. M., Huang, K. F. & Tai, K. Nonlinear dynamics induced by external optical injection in semiconductor lasers. *Quantum and Semiclassical Optics: Journal of the European Optical Society Part B* **9**, 765–784 (1997). URL https://doi.org/10.1088%2F1355-5111%2F9%2F5%2F009.

[7] Zhang, L. & Chan, S.-C. Cascaded injection of semiconductor lasers in period-one oscillations for millimeter-wave generation. *Opt. Lett.* **44**, 4905–4908 (2019). URL http://ol.osa.org/abstract.cfm?URI=ol-44-19-4905.

[8] Henry, C. Theory of the linewidth of semiconductor lasers. *IEEE Journal of Quantum Electronics* **18**, 259–264 (1982). URL https://doi.org/10.1109/JQE.1982.1071522.

[9] Savchenkov, A. A., Matsko, A. B., Ilchenko, V. S., Yu, N. & Maleki, L. Whispering-gallery-mode resonators as frequency references. II. Stabilization. *J. Opt. Soc. Am. B* **24**, 2988–2997 (2007). URL http://josab.osa.org/abstract.cfm?URI=josab-24-12-2988.

[10] Huang, G. *et al.* Thermorefractive noise in silicon-nitride microresonators. *Phys. Rev. A* **99**, 061801 (2019). URL https://link.aps.org/doi/10.1103/PhysRevA.99.061801.

[11] Kondratiev, N. M. *et al.* Self-injection locking of a laser diode to a high-Q WGM microresonator. *Opt. Express* **25**, 28167–28178 (2017). URL http://www.opticsexpress.org/abstract.cfm?URI=oe-25-23-28167.

[12] Kondratiev, N. M. & Lobanov, V. E. Modulational instability and frequency combs in whispering-gallery-mode microresonators with backscattering. *Phys. Rev. A* **101**, 013816 (2020). URL https://link.aps.org/doi/10.1103/PhysRevA.101.013816.

[13] Matsko, A. B., Liang, W., Savchenkov, A. A. & Maleki, L. Chaotic dynamics of frequency combs generated with continuously pumped nonlinear microresonators. *Opt. Lett.* **38**, 525–527 (2013). URL http://ol.osa.org/abstract.cfm?URI=ol-38-4-525.

[14] Lucas, E. *et al.* Ultralow-noise photonic microwave synthesis using a soliton microcomb-based transfer oscillator. *Nature Communications* **11**, 1–8 (2020). URL https://www.nature.com/articles/s41467-019-14059-4.

[15] Liang, W. *et al.* High spectral purity Kerr frequency comb radio frequency photonic oscillator. *Nature Communications* **6**, 7957 (2015). URL https://doi.org/10.1038/ncomms8957.

[16] Yi, X. *et al.* Single-mode dispersive waves and soliton microcomb dynamics. *Nature Communications* **8**, 14869 (2017). URL https://doi.org/10.1038/ncomms14869.

[17] Yi, X., Yang, Q.-F., Yang, K. Y., Suh, M.-G. & Vahala, K. Soliton frequency comb at microwave rates in a high-Q silica microresonator. *Optica* **2**, 1078–1085 (2015). URL http://www.osapublishing.org/optica/abstract.cfm?URI=optica-2-12-1078.

[18] Johnson, A. R. *et al.* Chip-based frequency combs with sub-100 GHz repetition rates. *Opt. Lett.* **37**, 875–877 (2012). URL http://ol.osa.org/abstract.cfm?URI=ol-37-5-875.

[19] Huang, S. W. *et al.* A low-phase-noise 18 GHz Kerr frequency microcomb phase-locked over 65 THz. *Scientific Reports* **5**, 13355 (2015). URL https://doi.org/10.1038/srep13355.

[20] Xuan, Y. *et al.* High-Q silicon nitride microresonators exhibiting low-power frequency comb initiation. *Optica* **3**, 1171–1180 (2016). URL http://www.osapublishing.org/optica/abstract.cfm?URI=optica-3-11-1171.

[21] Liu, J. *et al.* Ultralow-power chip-based soliton microcombs for photonic integration. *Optica* **5**, 1347–1353 (2018). URL http://www.osapublishing.org/optica/abstract.cfm?URI=optica-5-10-1347.

[22] Liu, J. *et al.* Photonic microwave generation in the X- and K-band using integrated soliton microcombs. *Nature Photonics* **14**, 486–491 (2020). URL https://www.nature.com/articles/s41566-020-0617-x.

[23] Herr, T. *et al.* Temporal solitons in optical microresonators. *Nat. Photon.* **8**, 145–152 (2014). URL http://dx.doi.org/10.1038/nphoton.2013.343.

[24] Marin-Palomo, P. *et al.* Microresonator-based solitons for massively parallel coherent optical communications. *Nature* **546**, 274-279 (2017). URL https://doi.org/10.1038/nature22387.

[25] Xue, X. *et al.* Microresonator Kerr frequency combs with high conversion efficiency. *Laser & Photonics Reviews* **11**, 1600276 (2017). URL https://onlinelibrary.wiley.com/doi/abs/10.1002/lpor.201600276.

[26] Kim, B. Y. *et al.* Turn-key, high-efficiency Kerr comb source. *Opt. Lett.* **44**, 4475–4478 (2019). URL http://ol.osa.org/abstract.cfm?URI=ol-44-18-4475.

[27] Kondratiev, N. M., Voloshin, A. S., Lobanov, V. E. & Bilenko, I. A. Numerical modelling of WGM microresonator Kerr frequency combs in self-injection locking regime. In Broderick, N. G. R., Dudley, J. M. & Peacock, A. C. (eds.) *Nonlinear Optics and its Applications 2020*, vol. 11358, 60 – 67. International Society for Optics and Photonics (SPIE, 2020). URL https://doi.org/10.1117/12.2555863.
